# Association of vaspin rs2236242 gene polymorphism with serum vaspin level, insulin resistance and diabetes in an Iranian diabetic/pre-diabetic population

**DOI:** 10.5937/jomb0-24671

**Published:** 2021-01-26

**Authors:** Maria Hosseini, Masoumeh Nezhadali, Mehdi Hedayati

**Affiliations:** 1 Islamic Azad University, Islamshahr Branch, Department of Biology, Islamshahr, Iran; 2 Shahid Beheshti University of Medical Science, Research Institute for Endocrine Science, Cellular and Molecular Endocrine Research Center, Tehran, Iran

**Keywords:** insulin resistance, diabetes mellitus type 2, vaspin, pre-diabetic, polymorphism, predijabetični polimorfizam, vaspin, dijabetes melitus tip 2, insulinska rezistencija

## Abstract

**Background:**

In recent years, the role of vaspin as an insulin-sensitizer has been studied widely. This is the investigation that examined the association of vaspin polymorphism rs2236242 on the vaspin level and the risk of type 2 diabetes and insulin-resistant Iranian pre-diabetic/diabetic population.

**Methods:**

A case-control study was conducted on 160 participants includes 80 participants holding (FBG) fasting blood glucose 3.88-5.55 (mmol/L) in the normal group, and 80 participants holding FBG≥5.55 (mmol/L) in a diabetic/pre-diabetic group. The serum vaspin and insulin were determined with ELISA (enzyme-linked assay) and biochemical variables by standard method. Tetra arms amplification system for the vaspin gene was performed. Statistical analysis was done using SPSS software version 20.

**Results:**

The means of age, body mass index (BMI), waist circumference (WC), hip circumference (HC), FBG, and vaspin were significantly different between normal and type 2 diabetic/impaired fasting blood group (P-value<0.05). rs2236242 showed association with Hip circumference (P-value<0.05). A significant association between allele A of rs2236242 with type 2 diabetes was seen (P-value<0.001). The vaspin levels showed a negative correlation with FBG (r =-0.296, P=0.001).

**Conclusions:**

Allele A of rs2236242 is a protective risk for type 2 diabetes, but no association of rs2236242 with insulin resistance was seen. The lower level of vaspin is a predictor for the progression of type 2 diabetes.

## Abstract (sr)

**Uvod:**

Poslednjih godina dosta je proučavana uloga vaspina kao senzitizatora insulina. Ovo istraživanje je proučavalo povezanost polimorfizma vaspina rs2236242 na nivou vaspina i rizik od dijabetesa tipa 2 kod iranske populacije predijabetičara i dijabetičara rezistentne na insulin.

**Metode:**

Sprovedena studija je uključivala 160 učesnika. Kod 80 učesnika nivo glukoze u krvi je bio 3,88-5,55 mmol/L - kontrolna grupa koja nije uzimala hranu pre davanja uzoraka (FBG), i 80 učesnika kod kojih je nivo FBG bio ≥ 5,55 mmol/L - grupa dijabetičara / predijabetičara). Serumski vaspin i insulin određeni su ELISA testom i biohemijskim varijablama standardnom metodom. Primenjen je tetra arms sistem za pojačavanje gena vaspina. Statistička analiza je urađena uz pomoć softvera SPSS, verzija 20.

**Rezultati:**

Srednje vrednosti životne dobi, indeksa telesne mase (BMI), obima struka (WC), obima kuka (HC), FBG i vaspina su se značajno razlikovale između kontrolne grupe sa normalnim vrednostima i vrednosti u grupi dijabetičara tipa 2 (P vrednost < 0,05). rs2236242 je pokazao povezanost s obimom kuka (P-vrednost < 0,05). Primećena je značajna povezanost alela A rs2236242 sa dijabetesom tipa 2 (P-vrednost < 0,001). Nivoi vaspina pokazali su negativnu korelaciju sa FBG-om (r = -0,296, P = 0,001).

**Zaključak:**

Alel A rs2236242 je zaštitni rizik za dijabetes tipa 2, ali nije primećena povezanost rs2236242 sa rezistencijom na insulin. Niži nivo vaspina je prediktor za progresiju dijabetesa tipa 2.

## Introduction

A considerable number of people who suffer from diabetes are type 2 diabetic patients. This issue properly shows how much intensive care and knowledge needs to clarify the different aspect of disease [1]. Type 2 diabetes mellitus (T2DM) is a multifactorial disorder that contributes to the involvement of many organs as a result of the development of the disease ranging from retinopathy, neuropathy, nephropathy, and cardiovascular disease that cause life-long vision lost, amputation, kidney disease and heart problems [2]. The death of beta-cell mass that occurs in diabetes mainly happens due to increased secretion of insulin hormone as a result of insulin resistance [3]. Pre-diabetes is also referred to as a condition where patients have high blood glucose, but this level does not reach high enough to categorize in the diabetic group. Pre-diabetics have an increased risk of T2DM than healthy people and are more capable of becoming diabetic population in the future [4]. The early diagnosis of the disease, which helps administer effective and immediate therapies, decreases the rate of morbidity and development of future complications [5]. In recent years, lots of attention has been paid to adipose tissues for their active endocrine role more than their energy storage role. Adipose tissue is synthesized proteins with hormonal properties [6]. Vaspin is one of the members of its family that was initially founded in Otsuka Long-Evans Tokushima Fatty (OLETF) rat visceral adipose. This serine protease inhibitor is a member of the serine protease family and assumed to have anti-inflammatory effects. Based on previous studies, it is assumed that vaspin acts as an insulin-sensitizer in response to a decrease in insulin sensitivity [7]. Some studies have shown that vaspin level is higher in diabetic and pre-diabetic patients compared to normal people [8] and the association between vaspin and obesity, diabetes, PCOS (Polycystic ovary syndrome), and insulin resistance was also confirmed in some studies [9] [7] [10]. Other research showed that vaspin is lower in cardiovascular disease but also served vaspin as a strong biomarker for cardiovascular disease [11]. Other study does not indicate a significant difference between vaspin levels in T2DM and nondiabetics group [12] and also in participants with or without renal insufficiency [13]. A single nucleotide polymorphism located in intron 4 of the vaspin gene, vaspin rs2236242 was found to be strongly associated with diabetes [7]. The association of allele A of vaspin rs2236242 gene polymorphism with T2DM and coronary artery disease (CAD) was found in China [14] [15] while other studies indicate no association of vaspin genotypes with diabetes [9]. Regarding these contradicting results, this study has been done to evaluate the association of rs2236242 of vaspin gene with type 2 diabetes, insulin resistance, serum vaspin level, and biochemical variables in healthy and pre-diabetic participants in Iran.

## Materials and Methods

### Study population

This case-control study was conducted on 160 participants. It included 80 participants having FBG 3.88-5.55 (mmol/L) in a non-diabetic group (ND), and 80 participants having FBG≥5.55 (mmol/L) in IFG/T2DM group (impaired fasting glucose/type 2 diabetic), aged 25-70 who referred to hospitals of Booali and Pars in Tehran, and Zanjan. The patients having FBG 5.5-6.94 (mmol/L) were regarded as pre-diabetics. Because of our strict exclusion criteria, we merged two groups of IFG and T2DM to have more study power. The participants were diagnosed by two specialists in the field, based on the protocol of the expert diabetes team [16]. The quantity of 10 mL blood sample was taken after overnight fasting of at least 12-14 hours [11]. 5 mL was kept in tubes contains EDTA (20 mg), and 5 mL was kept in tubes without EDTA. The participants were excluded from our study in cases of addiction, pregnancy, smoking, alcohol usage, autoimmune disease, cancer, HIV and hepatitis, liver, kidney, and heart disease, using diabetic drugs of any kind and other known diseases. The study was approved by the ethics committee of Islamic Azad University, Tehran Medical, according to the Helsinki declaration, under the code of IR.IAU.TMU.REC.1396.285.

### Anthropometric measurements

Initially, the protocol of the study was explained to the participants, then the written consent and questionnaire regarding their personal information, diets, and other information were filled by every individual that was present upon request (the questionnaires were previously provided by doctors in the field of diabetes). After filling the written consent, height, weight, HC, and WC were measured for each person. BMI was calculated through dividing weight in kilograms by height in meters squared. Weight was measured using a calibrated balance in a standing position without shoes.

### DNA extraction and PCR amplification

Deoxyribonucleic acid (DNA) was extracted using the salting out method [17]. For any individual 5 mL of blood was collected in EDTA tubes. This method includes lysing of the cell with a detergent or a washer, protein removal with salt and protein kinase, and finally, sedimentation with ethanol. Initially, 5 mL of lysis buffer was added to the samples and mixed well. Afterwards, it was centrifuged at 2000g for 10 minutes. The supernatant was discarded carefully. What remained was WBC (white blood cells). This step was repeated 2-3 times. The cells were suspended in 600 µL of Tris-EDTA-SALT. Later, 100 µL of SDS 10% was added, and proteinase k and incubated overnight. In the next step, saturated NACL was added (one-third of the total volume) and thoroughly mixed. It was then centrifuged at 10 000 g for 10 minutes. The supernatant was separated and poured into another test tube. Isopropanol 100% was added equal with the solution volume and mixed with inversion until the DNA was observed. The DNA was transferred to a microtube and then was centrifuged for 2 min at 10 000 g. The supernatant was discarded, and 70% ethanol was added. Then the tubes were centrifuged, and the supernatant was discarded. After drying the microtube at room temperature, 200 µL of TE was added to dissolve the DNA.

Detection of vaspin rs2236242 polymorphism genotypes was performed using the tetra-amplification refractory mutation system Polymerase chain reaction (T-ARMS-PCR). In terms of performing T-ARMS-PCR reaction, the total mixture of 20 µL included 10 µL master mix, 1 µL genomic DNA 50-100, 1 µL inner forward primer, 1 µL inner reverse primer, 0.8 outer reverse primer, 0.8 outer forward primer, and 5.4 µL of diluted water. The samples were covered by mineral oil to prevent evaporation. Then the samples were centrifuged with at high speed for 1 minute and then placed in a thermal cycler machine. The program of thermal cycler machine was as follows: Initial denaturation lasted for 5 minutes at 95 °C, then 30 cycles for each cycle, denaturation at 95 °C for 30 seconds, annealing at 57.3 °C for 30 seconds and extension at 72 °C for 30 seconds. The last step included a final extension at 72 °C for 5 min. For determining the genotypes, polyacrylamide 8% gel electrophoresis was used.

T-ARMS-PCR for vaspin gene rs2236242 was used using four primers. The sequence of the primers used in PCR with the corresponding PCR product sizes vaspin rs2236242 gene polymorphism are shown in Table 1.

**Table 1:** The characteristics of primers and PCR product size for vaspin rs2236242 PCR, polymerase chain reaction; bp, base pair

Gene polymorphism	Location	Primer (sequence 5’ to 3’)			PCR product size
vaspin rs2236242	Intron 5		Inner	Outer	T allele 174 bpA allele 248 bp Control 374 bp
		Reverse Control 374 bp Forward	ACCATCTCTCTGGCTTCAGGCTTC GGAGGCAGACCAGGCACTAGAA	CACAGGGACCCAGGATAACTTGCAAGACGCCGCTTCTGTGCACT	

### Measurement of biochemical variables

The enzymatic colorimetric method was also applied for the measurement of serum glucose. The glucose oxidase enzyme oxidizes glucose to glucuronic acid and H_2_O_2_. For the measurement of TC (total cholesterol), cholesteryl ester is converted to cholesterol by cholesteryl ester hydrolase and then cholesterol oxidase oxide cholesterol to cholesterol-4-en-3-one and H_2_O_2_. The level of TG (triglyceride) was also measured using the enzyme colorimetric method. For TG measurement, lipoprotein lipase enzyme was broken down TG to glycerol and free fatty acids. Then glycerokinase enzyme phosphorylates glycerol to glycerol phosphate; glycerol phosphate oxidase enzyme also changed glycerol phosphate to dihydroxyacetone phosphate and H_2_O_2_. HDL-Cholesterol was also determined through enzymatic colorimetric assay. Before the measurement of HDL (high-density lipoprotein), the precipitation of apolipoprotein B containing lipoproteins was performed by phosphotungstic acid and magnesium ions. The colorimetric indicator in all the aforementioned analyses was quinoneimine, which is prepared from 4-aminoantipyrine and phenol by H_2_O_2_ and is measured at 546 nm. LDL-C (low-density lipoprotein-cholesterol) concentration were calculated by Friedewald equation: [LDL-cholesterol] = [total cholesterol] − [HDL-cholesterol ] − [triacylglycerol/5] [18].

Calculation of insulin resistance and homeostatic model assessment of insulin resistance (HOMA-IR) was done by HOMA-IR=fasting insulin (mU/L) × fasting plasma sugar (mmol/L)/22.5. The quantity of a 5 mL blood sample was used for biochemical analysis.

Measuring of vaspin levels was performed by Sandwich enzyme-linked immunosorbent assay (ELISA) kits (ZellBio, Germany), and the result was read by DANA ELISA reader in 450 nm. Measuring of insulin hormone was also done by ELISA kits (Mercodia, Sweden) and also read by DANA ELISA reader. ELISA method performed by a purified immunoglobulin G (IgG) against a specific determinant on human adiponectin molecule and a mouse anti-adiponectin antibody conjugated to horseradish peroxidase (HRP) was used for detection.

### Statistical analysis

Statistical analysis was carried out using the SPSS software version 20 (SPSS, Chicago, IL, USA). Continuous variables were illustrated as mean ± SD (standard deviation), and categorical variables were expressed as number (percentage). Quantitative variables by the Kolmogorov-Smirnov test were studied in terms of normality condition. In cases when the distribution of some variables was not normal, the Mann-Whitney test was used for comparison of the case and normal group, and otherwise, the Independent T-test was used. Initially, biochemical and anthropometric parameters of the participants were analyzed by the frequency distribution table. For analyzing the study hypothesis, the Mann-Whitney test or independent Ttest was used. Qualitative variables were analyzed by Chi-square or Fisher's test. A logistic regression model was applied to estimate the odds ratio (OR) of vaspin rs2236242 genotypes with diabetes. The relationships between vaspin and other variables were presented as Spearman's rank correlation coefficients. The significance level was considered as P-value<0.05.

## Results

**Figure 1:**
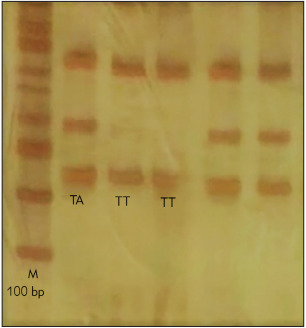
The location and length of T-ARMS-PCR for rs2236242

The result obtained from the electrophoresis of PCR products carried polymorphism rs2236242 is provided in Figure 1. M is 100 base pair ladder; T allele 174 bp; A allele 248 bp; Control band 374 bp. TA genotype with 3 bonds with the length of 174, 248, 374 bp, TT genotype with 2 bonds with the lengths of 174, 374 bp, and AA genotype with 2 bonds with the lengths of 248, and 374 bp is specified.

The study populations comprised IFG/T2DM and the ND group and are presented in Table 2. The result shows that the means of insulin, Cholesterol, HDL, and LDL was not statistically significant between the two groups (P-value>0.05). IFG/T2DM group had significantly higher mean levels of age, BMI, HC, TG, and FBG than those of the ND group (P-value<0.05). IFG/T2DM group also showed a significantly lower level of vaspin when compared with those in the ND group.

**Table 2:** Clinical characteristics of the study population Statistically significant P-value ≤0.05 IFG, impaired fasting glucose; T2DM, people with type 2 diabetes; ND, non-diabetics (healthy); BMI, body mass index; WC, waist circumference; HC, hip circumference; HDL, High- Density Lipoprotein; LDL, Low-Density Lipoprotein; TC, Total Cholesterol; TG, triglyceride; FBG, fasting blood sugar; IFG/T2DM, impaired fasting glucose/type 2 diabetics; ND, non-diabetic.

Variable	IFGT2DM (n=80)	ND	P-value
Age (years)	52.9±13.1	36.9 ±11.7	<0.001
BMI (kg/m^2^)	27.4 (24.8–29.8)	24.2 (21.5–27.3)	<0.001*
WC (cm)	96.3±12.6	103.1±13.1	0.006*
HC (cm)	105.2±14.6	96.9±15.7	0.004*
HDL (mmol/L)	1.19 (0.88–1.40)	1.21 (1.09–1.47)	0.232
LDL (mmol/L)	2.98±1.09	2.70±0/60	0.143
TG (mmol/L)	1.56 (1.11–2.28)	2.28 (0.94–1.82)	0.012*
TC (mmol/L)	4.78±1.27	4.31±1.04	0.055
FBG (mmol/L)	8.10 (6.83–9.99)	4.91 (4.55–5.22)	<0.001*
Insulin (pmol/L)	65.98 (42.36–106.26)	58.34 (38.20–96.53)	0.219
Vaspin (ng/mL)	0.98 (0.77–1.15)	1.43 (0.90–1.62)	<0.001*

Association of polymorphism vaspin rs2236242 with biochemical variables in Table 3 shows that vaspin genotypes are not associated with any of metabolic traits except hip-circumference (P-value<0.05).

**Table 3:** The distribution of studied variables in each genotype of rs2236242 BMI, body mass index; WC, waist circumference; HC, hip circumference; HDL, High-Density Lipoprotein; LDL, Low-Density Lipoprotein; TC, Total Cholesterol; TG, triglyceride; FBG, fasting blood glucose

Variable	TT	TA	P-value
Age (years)	45.13±14.4	45.76±14.18	0.826
BMI (kg/m^2^)	25.97±5.52	27.32±5.27	0.157
WC (cm)	100.04±12.9	100.23±11.28	0.938
HC (cm)	98.33±12.76	105.54±17.56	0.02*
HDL (mmol/L)	1.35±0.96	1.35±0.54	0.987
LDL (mmol/L)	2.83±0.76	2.85±091	0.905
TG (mmol/L)	1.67 (1.09–2.30)	1.14 (0.99–1.83)	0.065
TC (mmol/L)	4.72±1.21	4.47±1.08	0.329
FBG (mmol/L)	6.77±2.96	6.76±3.03	0.986
Insulin (pmol/L)	60.42 (43.06–105.56)	62.43 (36.81–104.86)	0.331
Vaspin (ng/mL)	1.01 (0.78–1.41)	1.05 (0.78–1.45)	0.835

The result in Table 4 shows the frequency of alleles and genotypes for rs223642 of vaspin gene polymorphism. However, there was an estimate of 18 missing pieces of data of vaspin rs2236242 gene polymorphism. The frequency of AA genotype was 0% in diabetic cases and 1.3% in non-diabetics. Therefore, OR was not calculated for this genotype. AA genotype was ignored, and analysis was performed again. The result showed that there is no statistically significant association in genotypes of rs2236242 between IFG/T2DM and ND group. It was also evident that the frequency of allele A is higher in ND (normal) compared with IFG/T2DM group, and this association was statistically significant (P-value<0.05). It shows that allele A of SNP 2236242 decreases the risk of type 2 diabetes in participants. No association of rs2236242 genotypes with insulin resistance was seen in the current study.

**Table 4:** Distribution of ADIPOQ genotypes of rs2236242 among T2DM and insulin-resistant sub-groups T2DM, type 2 diabetes; ND, non-diabetics; HOMA<2.24, insulin-sensitive; HOMA 2.24-3-59, interstitial insulin resistance; HOMA>3.59, insulin-resistant

Genotypes	T2DM	ND	OR (95%CI)	P-value
AA	0 (0)	1 (1.3)	-	-
TT	36 (57.1)	45 (57)	1.01 (0.52–1.97)	0.983
TA	27 (42.9)	33 (41.8)	1.04 (0.53–2.04)	0.897
A	27 (21.4)	35 (22.1)	4.67 (2–10.7)	<0.001*
Group	HOMA<2.24	HOMA 2.24–3.59	HOMA>3.59	P-value=0.812
TT	39 (57.4)	16 (53.3)	25 (61.0)	
TA	29 (42.6)	14 (46.7)	16 (39.0)	

The correlation of vaspin with parameters was shown in Table 5. A linear regression was applied in Table 5 for finding the correlation of parameters with vaspin. FBG and HC had a negative correlation with vaspin level.

**Table 5:** Spearman rank correlation coefficient of the vaspin with different clinical parameters BMI, body mass index; WC, waist circumference; HC, hip circumference; HDL, High-Density Lipoprotein; LDL, Low- Density Lipoprotein; TC, Total Cholesterol; TG, triglyceride; FBG, fasting blood sugar; HOM, homeostasis model assessment of insulin resistance

Quantitative variable	correlation	P-value
Age	-0.115	0.304
BMI	-0.128	0.17
WC	0.038	0.712
HC	-0.199	0.053*
HDL	-0.063	0.598
LDL	-0.115	0.365
CHOL	-0.106	0.353
FBG	-0.296	0.001*
Insulin	0.117	0.189
HOMA	-0.032	0.72

## Discussion

In this study, the association of rs2236242 gene polymorphism and its genotypes with the risk of type 2 diabetes and pre-diabetes, vaspin level, and biochemical variables has been studied. The current study revealed that the T2DM group had a significantly higher level of FBG, TG, HC, WC, age, and BMI than that in the normal group. FBG and TG levels were also higher in the T2DM group than in the normal in studies in China [19] and Egypt [20]. We found that vaspin level is significantly lower in T2DM than in the normal group, and this association is statistically significant and harmonizes with the study in Bangladesh [21] and India [22]. In this way, a study done on three categories of healthy participants, previously diabetic and newly diabetic in China, concluded that vaspin level is higher in healthy participants than in newly diagnosed diabetics, and vaspin is the least in previously diabetics than two other groups. It predicts that with the progression of diabetes, people show a lower level of vaspin [19]. Also, in China, a lower level of vaspin was found in the T2DM group than in ND, and vaspin level was recognized as a risk factor for new onset of T2DM along with the progression of diabetes [23]. Moreover, a decreased level of vaspin level was involved in the progression of diabetic nephropathy in 90 patients enrolled in a study in China [24]. These results are in accordance with our results. On the contrary, the research done in Iran-Zahedan, on 40 healthy participants and 40 patients from a diabetic group, a higher level of vaspin was found in T2DM than in the healthy group [25]. Also, another report from China on GDM patients and diabetic polyneuropathy patients showed a higher level of vaspin in diabetic patients than in a control group [26] [27]. Different results of different studies may come from diversity in ethnic features and the size of their sample that distinguish the populations from each other.

Our results also confirmed no significant association between rs2236242 genotypes and diabetes except for allele A of rs223642 that showed more frequency in control than the T2DM/IFG group (P-value<0.05). It shows that A allele of vaspin rs2236242 plays a protective role against type 2 diabetes. Allele A of SNP 223642 also played a protective role against diabetes among Egyptian women [23] and Chinese [28]. Allele A of rs2236242 also plays a protective role against obesity in Iran [29]. We did not find Homozygous state of A in T2DM and only one case in ND, so assessment of homozygous effect was not possible. In contrast, in German population AA genotype of SNP rs2236242 increased the risk of type 2 diabetes [30], and Chinese people also showed an association of A allele with diabetes, which means allele A increases the risk of T2DM [14]. There was no association between genotypes and allele frequencies with vaspin level in the current study as it was also found in Pakistan [9] and Egypt [7], which are constituent with our findings. We also found no association between genotype and allele frequencies with insulin resistance. In contrast, there was a close relation between serum vaspin and insulin resistance in Japanese subjects [31], and insulin resistance has an influence on the correlations between changes in serum vaspin concentration [32]. A significant positive correlation between vaspin and HOMA-IR was also seen in studies done in Iran [10] [33].

The correlation between serum vaspin level and markers of glucose metabolism and obesity is still controversial. In the current study, a negative correlation of vaspin with HC was seen as it was also seen in previous studies [22]. Vaspin had a negative correlation with FBG that contradicts with the study done on 40 diabetic and healthy participants in Iran [25]. In China, the FBG level also had a correlation with vaspin, which showed that vaspin is associated with the glucose indicators in different regions [24].

## Conclusion

The current study confirmed that vaspin rs2236242 is an influential factor of T2DM and affects metabolic parameters. T2DM patients had lower vaspin levels, which were not affected by different genotypes of rs1501299 in the present study. However, no association of this SNP with insulin resistance was seen in our research. Further studies with a larger sample size are needed to clarify the contradictions.

## Acknowledgements

We declare special thanks from Endocrine research center of ShahidBeheshti University particularly the head of center for their technical and scientific support in any circumstances. We also appreciate two specialists in the field of diabetes: Dr. Laleh Ghanei, Endocrinologist and Metabolism Specialist and member of faculty in Tehran Medical University and Dr Mehran Zaman Zadeh (member of American Diabetes Association (ADA) and also from the laboratory stuff of Booali hospital for the process of sample collection. All the authors read and approved the final version of manuscript. This research was not supported by any specific grant from public funding agency, commercial or not-for-profit sector.

## Conflict of interest statement

All the authors declare that they have no conflict of interest in this work.

## 
